# Estimating Annual CO_2_ Flux for Lutjewad Station Using Three Different Gap-Filling Techniques

**DOI:** 10.1100/2012/842893

**Published:** 2012-04-01

**Authors:** Carmelia M. Dragomir, Wim Klaassen, Mirela Voiculescu, Lucian P. Georgescu, Sander van der Laan

**Affiliations:** ^1^European Centre of Excellence for the Environment, Faculty of Sciences, Dunarea de Jos University of Galati, Street Domneasca No. 111, 800201 Galati, Romania; ^2^Centre for Isotope Research, University of Groningen, Nijenborgh 4, 9747 AG Groningen, The Netherlands; ^3^Climate and Environmental Physics, Physics Institute, University of Bern, Sidlerstrasse 5, 3012 Bern, Switzerland

## Abstract

Long-term measurements of CO_2_ flux can be obtained using the eddy covariance technique, but these datasets are affected by gaps which hinder the estimation of robust long-term means and annual ecosystem exchanges. We compare results obtained using three gap-fill techniques: multiple regression (MR), multiple imputation (MI), and artificial neural networks (ANNs), applied to a one-year dataset of hourly CO_2_ flux measurements collected in Lutjewad, over a flat agriculture area near the Wadden Sea dike in the north of the Netherlands. The dataset was separated in two subsets: a learning and a validation set. The performances of gap-filling techniques were analysed by calculating statistical criteria: coefficient of determination (*R* ^2^), root mean square error (RMSE), mean absolute error (MAE), maximum absolute error (MaxAE), and mean square bias (MSB). The gap-fill accuracy is seasonally dependent, with better results in cold seasons. The highest accuracy is obtained using ANN technique which is also less sensitive to environmental/seasonal conditions. We argue that filling gaps directly on measured CO_2_ fluxes is more advantageous than the common method of filling gaps on calculated net ecosystem change, because ANN is an empirical method and smaller scatter is expected when gap filling is applied directly to measurements.

## 1. Introduction

A good knowledge of the production rate and local storage of CO_2_ as well as of the flow of energy (mainly heat and momentum) and mass (mainly gasses and vapour) is important in view of the recent reports about global climate change. The CO_2_ flux to and from the atmosphere is a measure of growth or decrease of biomass in an ecosystem. Inversely, ecosystem-atmosphere gas fluxes can be modelled using knowledge of biomass changes. 

When atmospheric conditions (relative humidity, wind velocity, air temperature, and global radiation) are constant and the main vegetation is homogeneous and situated on a flat terrain for an extended distance upwind, the eddy covariance method is the most reliable for determining the quantity of CO_2_ exchange between the biosphere and atmosphere [1]. When the eddy covariance method is used over natural and complex landscapes or during atmospheric conditions that vary with time, the measurements must include estimates of atmospheric storage, flux divergence, and advection. Gross ecosystem carbon uptake and ecosystem respiration are the two major components of NEE with the atmosphere [2]; thus, the exchange of CO_2_ between the atmosphere and the biosphere is the balance between the gross ecosystem productivity and ecosystem respiration [3].

 Gaps or missing data originate in calibration errors, night-time air drainage flow beneath sensors, and missing data due to instrument failure or extreme weather conditions [4–8]. Calculating carbon balances from daily to annual time scales is a challenge because of these errors. Moreover, gaps in recorded data set are usually not distributed randomly during the year due to seasonal variations in the climate and ecosystem function, which adds difficulties to the gap filling process and data processing [9]. Another source of gaps is, for the particular data set used in this study, the selection of wind direction with flow over homogeneous vegetation, because data with northern wind are influenced by the nearby Wadden Sea.

 Gap-filling methods have developed starting with the innovative procedure of Falge [10]. Presently, gap-filling methods use interpolation, probabilistic filling, look-up tables, nonlinear regression, artificial neural networks, and process-based models in a data-assimilation mode [11–25].

Despite intensive studies of gap-filling techniques, there is a need to improve the quality and reliability of the results, which are still highly dependent on meteorological conditions, on methods, and on the characteristics of the data set. In this paper, we aim at disentangling between three methods that can be used for gap filling and to find the most reliable method that could be used for a set of given meteorological conditions, based on a defined set of available data. We compare results obtained with three methods: multiple regression (MR), artificial neuronal network (ANN), and multiple imputation (MI), which were applied to data basis consisting of hourly eddy covariance measurements collected from Lutjewad, The Netherlands, during 2008.

## 2. Data and Methods

### 2.1. Experimental Data

The measurements are taken at the 60-meter-tall atmospheric research tower Lutjewad (6°21′E, 53°24′N) located in the north of The Netherlands. Concentrations of several greenhouse gases [26] and their isotopes are measured on a continuous basis and by automated flask sampling techniques [27]. Meteorological data include wind speed, air temperature, solar radiation, and relative humidity, measured at various heights. The wind direction is measured at 60 meters, precipitation is measured at ground level, and atmospheric pressure is measured at 7 meters. Starting with the summer of 2006, a Gill Windmaster Pro 3d-sonic anemometer/LICOR LI-7500 infrared CO_2_ and H_2_O analyzer combination is running at a height of 50 meters. The collected raw EC data have a time resolution of 10 Hz. The 10 Hz data were processed to fluxes with AltEddy software, a powerful program written at AltTerra (Wageningen University and Research Centre). Hourly averaged data were used in this study because the turbulence has a low frequency at the height where the CO_2_ flux is measured [28]. Half-hourly averaged momentum fluxes were underestimated by 4.4% compared to hourly fluxes.

 The site is influenced mainly by winds originating from southwest and west (about 30% of the time) [29]. Directly to the north of the tower is a dike with a height of almost 8 meters, running in a direction of about 75° from north. To the north of these lie salt marshes of about 800 meters, followed by the tidal Wadden Sea of about 8 km, and the island of Schiermonnikoog, beyond which starts the North Sea. In all other directions, the region is dominated by arable land for fetches up to at least 10 km, mostly sown with grain, sugar beets, and potatoes. Because of the remoteness of the location, anthropogenic sources of CO_2_ apart from the arable land are rather small. Northern winds are influenced by fluxes from the salt marsh and Wadden Sea, and southern winds are influenced by the existence of arable crops. In order to avoid sea influences, we selected only wind direction from the agricultural area between 95° and 215°.

Each hourly averaged flux measurement is accompanied by a quality data factor from 1 to 10, where 1 denotes the highest quality [30]. Only data with quality factor 1 have been used in the present study. Due to factor quality and wind-based limitations, about 60% of the existing database is rejected. Most of the data with a high-quality factor have been found from June till September. The lowest number of high-quality measurements of CO_2_ flux appears to have been collected in November, when only 69 measurements were used, compared to an average of around 400 used for summer months.

### 2.2. Gap-Filling Methods

Gap filling in the CO_2_ atmospheric flux was done by three methods: MR, ANN, and MI, using the statistical software program SPSS (version 16 and 17 SPSS Inc., IL, USA). Hourly data, separated for each month, were used in the analysis, and monthly averages were calculated in order to account for seasonal changes in plant biomass and ecosystem exchange.

 These data were obtained as follows: all hourly high-quality measurements obtained during the whole year have been separated in two equal sets. The first half was kept as a witness dataset and was considered as unknown. These data were considered as missing, thus creating artificial gaps. The second half has been considered as learning data, that is, this dataset was considered “known” and was introduced as such in each of the three statistical gap-filling methods. This way we can compare the CO_2_ flux that was actually measured with the gap-filled CO_2_ flux obtained with the MI, ANN, and MI models, as if these measurements were not available.

 The gap fill methods were used directly on measured CO_2_ fluxes, in contrast to common methods that fill gaps in NEE [9, 10]. The difference between CO_2_ flux and NEE in our case is mainly due to CO_2_ storage between the surface and measurement height. Filling gaps directly in measured CO_2_ data has the advantage that the results are not deteriorated by additional scatter from inaccurate estimations of CO_2_ storage. This is especially important when measurements are executed at elevated height, where storage may be large. However, some differences between CO_2_ flux and NEE exist: CO_2_ fluxes depend on wind velocity, whereas NEE is supposed to be independent of such an atmospheric variable. Input values for all three gap fill methods in this study are meteorological parameters (air temperature, wind velocity, global radiation, and relative humidity) associated with output data, CO_2_ flux (Table 1). Wind velocity is closely related to friction velocity and has the advantage that its measurement is more reliable.

#### 2.2.1. Multiple Regression

We used a stepwise multiple regression analysis (MR) to predict missing data of CO_2_ flux using the hourly data measurements of high quality of flux and meteorological condition in SPSS software.

Meteorological parameters that influence CO_2_ fluxes are global radiation, wind velocity, relative humidity, and air temperature. Correlation coefficients between hourly CO_2_ flux and each of the four parameters have been calculated. The correlation coefficient *R* ^2^ between global radiation and CO_2_ flux is the highest, with a value of 0.96. The other correlation coefficients are 0.93 for wind velocity, 0.51 with humidity, and 0.21 with the temperature. The SSPS program works as follows: global radiation score is entered first as a basis variable, while wind velocity, relative humidity, and air temperature would count for the variance. Next, the algorithm evaluates whether the remaining variables contributed significantly to the *R* ^2^ (the part of the variance in the response variable that the explanatory variables account for). If they did not, they would not be entered in the equation, in spite of the fact that initially their correlation levels were about the same strength. Stepwise regressions can be done forward, backward, or both ways, and, in all cases, the computer picks the regression configuration based on purely statistical information, with no logical or theoretical assumptions involved [31].

Multiple regressions require a large number of observations, and the number of input variables must substantially exceed the number of predictor variables. The equation has the following form:

(1) $$\begin{array}{c} y=b_{1}x_{1}+b_{2}x_{2}+\cdots +b_{n}x_{n}+c, \end{array}$$

where *b*_1_, *b*_2_,…, *b*_*n*_ are regression coefficients. The standard input method is simultaneous because all variables are introduced into the equation at the same time. Each predictor is analysed and evaluated by the influence it has on the prediction of the dependent variable. Variables can be retained or deleted on the basis on the associated statistics [32].

#### 2.2.2. Artificial Neural Networks

Artificial neural networks (ANNs) are purely empirical nonlinear regression. ANNs are composed of nodes connected by weight that are the regression parameters [33–35]. The multilayer perceptron (MLP) is a feed-forward neural network architecture and uses different linear combination functions and nonlinear sigmoidal activation functions. The MLP architecture contains an input layer, at least one hidden layer and an output layer. Each unit from the input layer is connected to a unit from the second layer, and the output layer is connected to the hidden layer. The input units are used to predict the values of the target variable. The hidden units execute an internal nonlinear transformation, and the output units create predicted values and then back-propagate errors (compares the difference between the predicted values with the values of the output units) adjusting the weights so that the network output optimally approximates CO_2_ flux. In the present study, a network with one hidden layer was used.

The input values pass the network, and the error is calculated by a comparison of the network's outputs *y*_*j*_ with measured target values *m*_*j*_. The quality of the network is evaluated on the basis of the mean squared error (MSE). *E* is the error as accumulated over all *N* data records that served as learning patterns [36]:

(2) $$E=\frac{1}{N}\overset{N}{\underset{j=1}{\sum }}{\left(m_{j}-y_{j}\right)}^{2}.$$

#### 2.2.3. Multiple Imputation (MI)

Multiple imputation (MI) uses a Markov Chain Monte Carlo algorithm to replace missing value with a range of estimated (imputed) values for each missing item. MI uses a regression model to predict missing values. The MI technique consists of three steps: imputation, analysis, and pooling. First, sets of plausible values for missing data are created that reflect uncertainty about the estimated model. Each of these sets of plausible values is used to impute the missing values and to obtain a complete data set. Second, each of these data sets is analyzed using statistical methods. Third, the results are combined, which allows the uncertainty regarding the imputation to be taken into account [37]. The observed values are the same as in the original data set, and only the missing values have different estimated values [16, 38].

 The accuracy of MI can be improved by using the multiple imputations (5–10 imputations) instead of the single imputation method that underestimates the error variance of missing data [39].

### 2.3. Statistical Performance Measures

Five performance indicators were calculated for describing the accuracy of the three gap-filling methods: the mean square bias (MSB), maximum absolute error (MaxAE), and mean absolute error (MAE), which calculate the magnitude and distribution of individual errors, and root mean square error (RMSE), *R*-squared (*R* ^2^), which measures the correlation.

MSB is used to evaluate the performance of an estimator and is given by

(3) $$\text{MSB}=\frac{1}{N}\overset{N}{\underset{i=1}{\sum }}{\left(p_{i}\left(x_{i},t\right)-o_{i}\left(x_{i},t\right)\right)}^{2},$$

where *o*_*i*_ is individual observed CO_2_ flux data, *p*_*i*_ is the predicted values, *N* equals the number of hourly predicted observation pairs.

MaxAE represents the largest forecasted error, expressed in the same units as the dependent series. MaxAE is useful for analyzing the worst-case scenario for forecasts data:

(4) $$\text{MaxAE}=\max\left(|p_{i}-o_{i}|\right).$$

MAE measures how much the series varies from its model-predicted level:

(5) $$\text{MAE}=\frac{1}{N}\overset{N}{\underset{i=1}{\sum }}|p_{i}-o_{i}|.$$

RMSE is a measure of the difference between the observed and predicted CO_2_ flux data, and it was used to provide the average error of model:

(6) $$\text{RMSE}={\left(\frac{1}{N}\overset{N}{\underset{i=1}{\sum }}{\left[p_{i}-o_{i}\right]}^{2}\right)}^{1/2}.$$

*R* ^2^ was used to estimate the proportion of the total variation in the series that is explained by the model:

(7) $$R^{2}=\frac{{\left\{\sum \left(p_{i}-\bar{p}\right)\left(o_{i}-\bar{o}\right)\right\}}^{2}}{\sum {\left(p_{i}-\bar{p}\right)}^{2}\sum {\left(o_{i}-\bar{o}\right)}^{2}},$$

where *o*_*i*_ is individual observed CO_2_ flux data, *p*_*i*_ the predicted values, $\bar{p}$ and $\bar{o}\;$their means. 

## 3. Results and Discussion

### 3.1. Diurnal Variation

**Figure 1:**
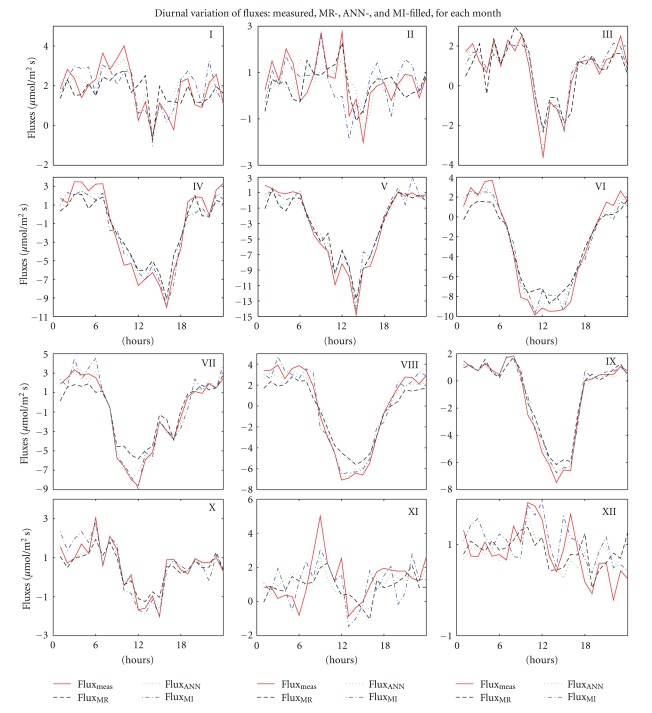
Diurnal variation of the four fluxes for each month, represented as follows: measured flux: red, continuous line; MR filled: black, dash line; ANN filled: green, dotted line; MI filled: blue, dash line. The month is identified by corresponding roman numerals in the right corner of each plot.

Diurnal variations of measured and gap-filled hourly fluxes are shown in Figure 1 for each month. The diurnal cycle of the measured flux from March to October is nicely reproduced by all three methods, but MR and MI underestimate the negative peak during daytime, especially during the summer months. Higher differences between measured and gap-filled data seem to occur in daytime, when the average CO_2_ flux is negative and large, compared to the night time, when CO_2_ flux is positive and small. In all months, the ANN line is the closest to the measured values, suggesting that the ANN method gives more accurate results than MR and MI.

 The highest differences are observed in November, when an hourly mean of the measured flux (at 9 AM) is about 2 times higher than the gap-filled data. Further analysis showed that the value at 9 AM is the average of only two measurements, so all random errors are already included in this value.

### 3.2. Seasonal Effects

**Figure 2:**
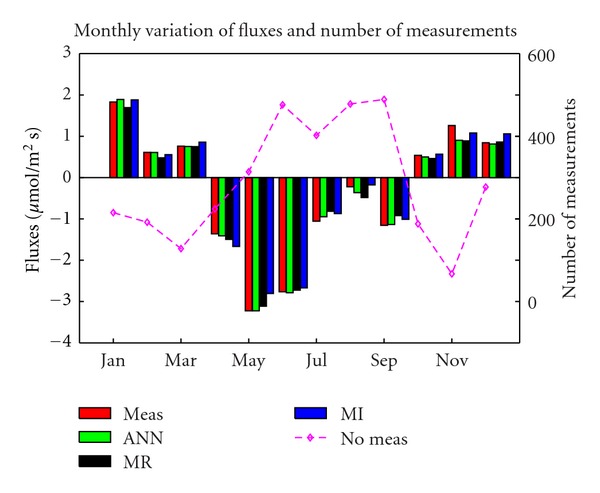
Monthly variations of the measured CO_2_ flux (red) and of the CO_2_ flux gap filled by multiple regression (MR: black), artificial neural network (ANN: green), and multiple imputation (MI: blue). Number of measurements is also shown as a pink dotted line.

The results for monthly averages of gap-filled CO_2_ fluxes are shown in Figure 2, together with measured values. The plot shows that, most of the time, gap-filled values are close to the measured ones. However, some departing results can be noticed, for instance, in July, August for MR, or April for MI. The highest difference between measured and all gap-filled data exists in November.

Data were grouped by season in order to observe a possible seasonal effect on the accuracy of gap-filling results. Winter seasons consist of November–February, March, April, September, October are considered to be part of the equinox season, while summer months are May–August.

In winter, there is less sunlight and air temperature is lower. Most fields are bare except maybe for winter wheat with some above ground biomass, so photosynthesis is expected to be very small and soil respiration may explain the positive CO_2_ flux. The highest positive CO_2_ fluxes are observed in January. The averages of measured and gap-fill data of CO_2_ flux for the entire cold season (January, February, November, and December) are presented in Table 2. The ANN method is the closest to the measured flux, with differences between gap-filled and observed data ranging from −0.06 to 0.03 *μ*mol m^−2^ s^−1^ (except November). MR differences are between 0.02 to 0.14 *μ*mol m^−2^ s^−1^, and MI differences range from −0.18 to 0.05 *μ*mol m^−2^ s^−1^. Concluding, all gap-filled methods provide good results for winter time. 

 The equinoctial season consists of data from March, April, September, and October. The equinoctial average of the measured and gap-fill data of CO_2_ flux is presented in Table 3. Differences range from 0.31 to −0.42 *μ*mol m^−2^ s^−1^ for MI, while ANN departs from measured results with values from −0.02 *μ*mol m^−2^ s^−1^ to 0.05 *μ*mol m^−2^ s^−1^. Differences between MR-gap-filled and measured data are in between, varying from –0.23 *μ*mol m^−2^ s^−1^ to 0.14 *μ*mol m^−2^ s^−1^. Concluding, the best fill for equinox months is obtained using the ANN method.

 The warm season is considered to last from May to August. The average of measured CO_2_ flux and gap-fill data by MR, ANN, MI is presented in Table 4. Again, ANN gives the best results out of the three methods for the warm season as well. 

Atmospheric conditions and the assimilation/respiration of plants change with the seasons. During the summer season, the absolute value of the CO_2_ flux is higher because photosynthesis occurs most rapidly in summer, thus CO_2_ exchange is more intense. Peaks of the negative CO_2_ flux in May and June might be explained by an increased uptake of the vegetation in the growing season. Such effects of the vegetation during the main growing season are reported in other studies of CO_2_ fluxes [36]. Also, respiration increases with temperature.

### 3.3. Statistical Analysis

**Figure 3:**
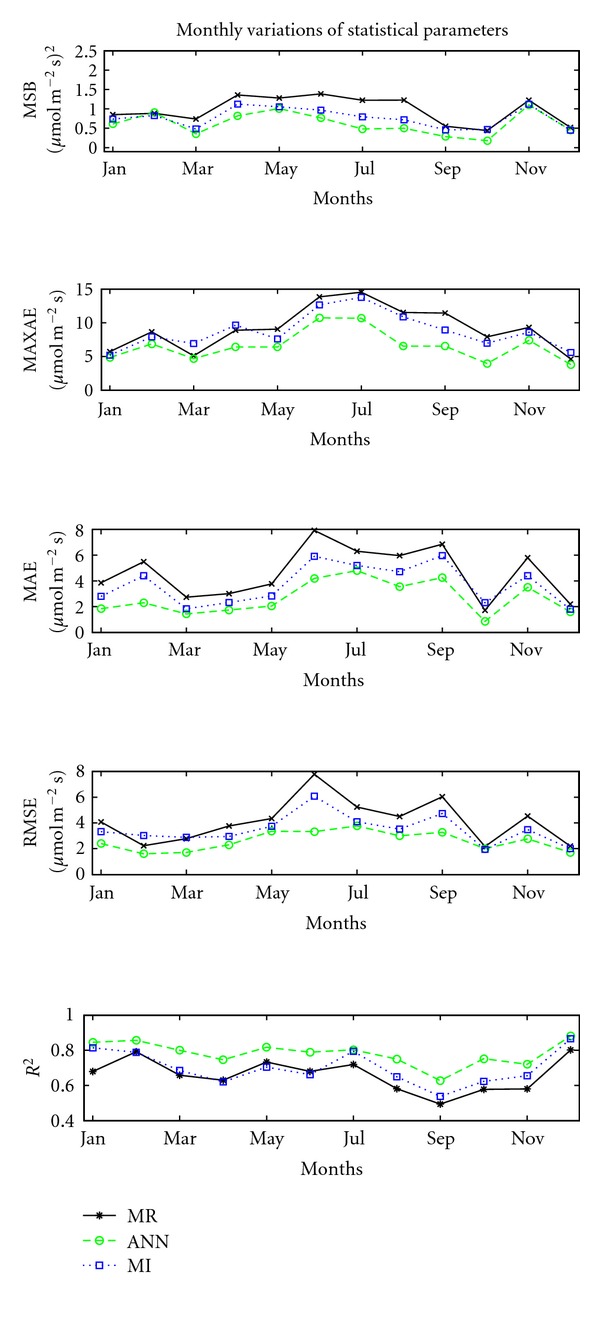
Monthly variation of the performance of gap-filling techniques MR (black line, stars), ANN (green line, circles) and MI (blue line, squares) measured by statistical parameters.

In order to have a better view of the accuracy of each gap-fill method, we compared the monthly variation of MR, ANN, and MI using five statistical parameters (Figure 3). The MSB is a handy criterion for the evaluation of the gap-filling techniques, determining the systematic error for a long dataset. Its monthly value is computed by the average square root of biases for every hour of the day during a specific month. Averaging over all observations during a month of a certain time of day reduces the impact of statistical uncertainties in the measurements and thus should give a better focus of the performance of the gap filling method but less focus on the uncertainty in observations. 

Higher biases are observed in summer, especially for the MR method. Lower differences between measurements and gap fill occur in winter, except for November, when the mean square bias is high for all three methods. The best results are obtained at equinox, when day and night periods are of similar length and CO_2_ uptake during day time is almost equal by release during the night. ANN performs better than MI every month and better than MR in 11 out of 12 months. A clear seasonal effect is seen in the MR and MI methods: the poor result of MR and MI during months with high irradiation might be partly caused by the use of linear regression on irradiation. In reality, photosynthesis saturates with high irradiation because the vegetation cannot photosynthesize more quickly. This might indicate that MR and MI calculations are dominated by the radiation monthly budget: the method gives poor results when the radiation is high and good results when the radiation is low. 

One explanation for the poor result in November might be the fact that the amount of data used for gap-fill is too small (only 69 measurements) to give reliable results, regardless of gap-fill method. Figure 3 shows that results are good in March, although the number of data that has been used is also relatively small (128, compared to 200–500 for the rest of months). On the other hand, the flux in March is 60% from the November one. This might suggest that any gap-fill method will become unreliable when the number of high-quality measurements is below a certain threshold. 

MaxAE confirms the results described above, with a higher accuracy for all gap-filling techniques for autumn and winter months (except November) and a lower accuracy for June and July. MAE measures the average magnitude of the errors in a set of forecasts, and the same pattern was obtained for all three gap filling methods. The best performance is given by ANN in equinox months, with a MAE of 0.86 *μ*mol m^−2^ s^−1^ in October. Low values of RMSE indicate a good fit of the model to measurements. Again, ANN is overall the best method while the worst gap-fill method is the MR method. 

The highest values of *R* ^2^ are obtained for ANN, supporting that, indeed, ANN is the best gap-fill technique. All methods give good results in December for all three methods, with *R* ^2^ ranging from 0.80 (MR) to 0.88 (ANN). A low accuracy of all methods is seen in September, when the lowest *R* ^2^ is 0.49 (MR) and the highest is 0.63 for ANN. 

A concise view of gap-filling methods is given in Table 5, where annual averages of all statistical parameters is given for all three gap-filling techniques. Bold digits show the best result, and it is clear that the best response at each statistical test is given by the ANN method. Only meteorological parameters have been considered in our calculations; thus, it might be necessary to take into account some indicators of specific spring biological processes. The large bias in April, compared to other equinox months, might be due to the fact that the CO_2_ uptake by the agriculture area is strong or to the fact that large differences exist in the CO_2_ uptake between day and night. Good results are obtained in autumn which might be explained by the fact that effects of global radiation and air temperature on ecosystem cause the reduction of the potential for agriculture area carbon sequestration. 

Correlation coefficients between biases (MSB) of each gap-fill method and each meteorological parameter are given in Table 6, which shows that the ANN method is independent of the meteorological parameters. The performance of MR gap-fill method is strongly influenced by the global radiation and by the relative humidity, while the performance of MI depends on relative humidity. None of the three methods is sensitive to the number of measurements, although a possible limitation to a minimum number might be necessary for getting reliable results (see the case of November). 

To find which statistical indicator is the best for the determination of the optimal gap fill method, we add a test. Based on the above results, that is, that ANN in the best gap-fill technique, we calculated the difference between other methods and ANN and the scatter in that difference, measured by the variance. The ratio between scatter and the mean difference is a measure of the probability that ANN is actually better than another method. The ratio between mean and scatter is a measure of the probability that the results are caused by statistical uncertainty; the larger the number, the smaller the probability. 

The results of the test, shown in Table 7, show that the largest ratio is found for *R*-squared for the difference between MR and ANN, but this is not valid for the difference between MI and ANN, for which MaxAE seems to have the highest value. However, MaxAE, *R* ^2^, and MAE are very close, without any clear difference. All ratios are relatively close to each other, suggesting that all indicators can be used in evaluating the relative performance of a method compared to others.

## 4. Conclusions

Three gap-filling methods, MR, ANN, and MI, were used for estimating atmospheric CO_2_ flux, and their accuracy was studied using an hourly dataset covering one whole year (2008) from an agricultural area. Poor weather and/or northern wind conditions led to large gaps in data. Errors may be introduced also by a nonrandom distribution of data set gaps. Each of these statistical methods gives a good estimation of atmospheric CO_2_ flux, when number of gaps of original dataset was small and had a random distribution. The small biases that were found in this study imply that gap fill methods could be used directly on CO_2_ measurements. 

The first general conclusion is that, overall, ANN gives better results than MR and MI at yearly, monthly, and diurnal scale. ANN has hardly any diurnal variation, while the MR and MI methods perform better during night time than during day time. The ANN performance indicators are better for almost every month. The efficiency of the gap-filling methods depends on the season, especially for MR and MI. Higher biases are met during warm seasons (April–August), when CO_2_ fluxes are negative and their absolute values are higher. All three methods give low errors in colder seasons (September–March), when CO_2_ flux is positive and smaller. The decrease of biases towards August (especially in ANN results) coincides with a decrease in the absolute value of the CO_2_ flux. An exception occurred in November when very few high-quality measurements were available as learning dataset. We conclude that sufficiently high-quality measurements might be needed to reduce the impact of random errors in the results of the gap-fill methods and a minimum critical number of measurements is needed in order to reduce random errors and obtain reliable results. Negligible errors were obtained for the year average flux, but the good overall result for the MR and MI methods was caused by compensating errors in summer and winter and compensating errors in day and night time. 

 The ANN produced the best results, having the lowest annual average for RMSE and the highest *R* ^2^ values. The accuracy of the method has a small seasonal and diurnal variation, which means that this method is almost independent on environmental conditions. In contrast, the accuracy of the MR and MI methods varies significantly with the season and with the time of day. 

The ecosystem exchange with the atmosphere influenced the results of gap filling CO_2_ flux for each of the three methods. This is related to two causes: large difference in CO_2_ exchange during day and night and strong temporal change throughout a month because increasing soil cover of the vegetation. Since only meteorological parameters have been considered in the calculations, this might be an indication that biological proxies should be also taken into account. 

Gaps were filled on CO_2_ measurements instead of NEE calculations because CO_2_ measurements lack additional noise from inaccurate storage measurements. A possible drawback of using CO_2_ data as input might be that relations between CO_2_ flux and biological processes are less clear compared to NEE data, but this argument does not hold when the empirical ANN method is used to fill gaps. 

Therefore, we conclude that all three methods could be used to calculate year-round average flux, but ANN is clearly preferred when shorter timescale data sets are analysed.

## Figures and Tables

**Table 1:** Input variables used for gap filling.

No.	Input variables	Unit	Instrument/note	Height
(1)	CO_2_ flux	*μ*mol m^−2^ s^−1^	Licor LI-7500 open path CO_2_-H_2_O sensor combined with a Gill Windmaster Pro 3D sonic anemometer	50 meters

(2)	Global radiation	Wm^−2^	Kipp CMA pyranometer	2 meters

(3)	Wind velocity	ms^−1^	Three-dimensional sonic anemometer-thermometer	40 meters

(4)	Relative humidity	%	Campbell HMP45C	40 meters

(5)	Air temperature	°C	PT-100 resistors	40 meters

**Table 2:** Monthly averages of the measured CO_2_ flux and of the CO_2_ flux gap filled MR, ANN, MI for cold season.

Month	Measured	MR	ANN	MI
	[*μ*mol m^−2^ s^−1^]	[*μ*mol m^−2^ s^−1^]	[*μ*mol m^−2^ s^−1^]	[*μ*mol m^−2^ s^−1^]
January	1.83	1.69	1.89	1.88
February	0.61	0.48	0.61	0.55
November	1.26	0.90	0.89	1.08
December	0.84	0.86	0.81	1.06
Average	**1.13**	**0.98**	**1.05**	**1.14**

**Table 3:** Monthly averages of the measured CO_2_ flux and of the CO_2_ flux gap filled MR, ANN, MI for equinoctial season.

Month	Measured	MR	ANN	MI
	(*μ*mol m^−2^ s^−1^)	(*μ*mol m^−2^ s^−1^)	(*μ*mol m^−2^ s^−1^)	(*μ*mol m^−2^ s^−1^)
March	0.76	0.75	0.75	0.86
April	−1.36	−1.50	−1.41	−1.67
September	−1.15	−0.92	−1.13	−1.01
October	0.54	0.46	0.50	0.56

Average	**−0.31**	**−0.30**	**−0.32**	**−0.31**

**Table 4:** Monthly averages of the measured CO_2_ flux and of the CO_2_ flux gap filled MR, ANN, MI for warm season.

Month	Measured	MR	ANN	MI
	(*μ*mol m^−2^ s^−1^)	(*μ*mol m^−2^ s^−1^)	(*μ*mol m^−2^ s^−1^)	(*μ*mol m^−2^ s^−1^)
May	−3.22	−3.11	−3.22	−2.80
June	−2.76	−2.72	−2.78	−2.67
July	−1.06	−0.82	−0.95	−0.87
August	−0.22	−0.48	−0.37	−0.18)

Average	**−1.82**	**−1.78**	**−1.83**	**−1.63**

**Table 5:** Yearly average of statistical performance for MR, ANN, and MI gap-filling techniques.

Statistical parameters	MR	ANN	MI
Mean bias (*μ*mol m^−2^ s^−1^)^2^	0.97	**0.62**	0.77
MaxAE (*μ*mol m^−2^ s^−1^)	9.21	**6.57**	8.72
MAE (*μ*mol m^−2^ s^−1^)	4.64	**2.68**	3.71
RMSE (*μ*mol m^−2^ s^−1^)	4.13	**2.60**	3.48
*R* ^2^	0.66	**0.78**	0.70

**Table 6:** Correlation coefficients between the biases (MSB) of the three gap-filling methods and meteorological parameters involved in the analysis. Values written in bold are significant at the 0.01 level.

Parameter	MR	ANN	MI
Global radiation	***0.65***	0.23	0.37
Air temperature	0.39	−0.19	0.39
Wind velocity	−0.04	0.05	0.16
Relative humidity	***−0.52***	−0.45	***0.51***
Number of measurements	0.20	−0.27	0.16

**Table 7:** Statistical parameters.

		MR-ANN	MI-ANN
MSB (*μ*mol m^−2^ s^−1^)^2^	Average	0.35	0.14
	Sigma (*n* − 1)	0.25	0.13
	**Ratio**	**1.41**	**1.11**

*R* ^2^	Average	−0.12	−0.08
	Sigma (*n* − 1)	0.04	0.04
	**Ratio**	**3.21**	**1.88**

RMSE (*μ*mol m^−2^ s^−1^)	Average	1.54	0.88
	Sigma (*n* − 1)	1.14	0.75
	**Ratio**	**1.35**	**1.17**

MAE (*μ*mol m^−2^ s^−1^)	Average	1.96	1.03
	Sigma (*n* − 1)	0.93	0.61
	**Ratio**	**2.09**	**1.70**

MaxAE (*μ*mol m^−2^ s^−1^)	Average	2.64	2.15
	Sigma (*n* − 1)	1.56	1.13
	**Ratio**	**1.69**	**1.90**
